# Supplementary material for: 24-nt reproductive phasiRNAs are broadly present in angiosperms
Source: Nat Commun. 2019 Feb 7;10:627. doi: 10.1038/s41467-019-08543-0 (PMC6367383; doi:10.1038/s41467-019-08543-0)
Supplement: Supplementary file 1 — Supplementary Information [file 41467_2019_8543_MOESM1_ESM.pdf]

Supplementary Information

24-nt reproductive phasiRNAs are broadly present in angiosperms

Rui Xia, Blake C. Meyers *et al.*

Supplementary Figures 1 to 8.

Supplementary Figure 1. miR2275/24-nt phasiRNA pathway components in litchi.
A. A cluster of three *MIR2275* precursors in scaffold598. The mature miR2275 and miR2275* sequences are marked in red and orange, respectively. Bulges within the duplexes are indicated by a cyan circle.
B. Three MIR2275 precursors are transcribed into a single transcript. sRNA and strand-specific RNA-seq data are viewed in different tracks in the Integrative Genomics Viewer (IGV).
C. An example 24-nt *PHAS* locus from litchi, showing validation of miR2275 cleavage in the PARE data and strongly phased 24-nt siRNAs, also viewed in IGV (as in panel B).

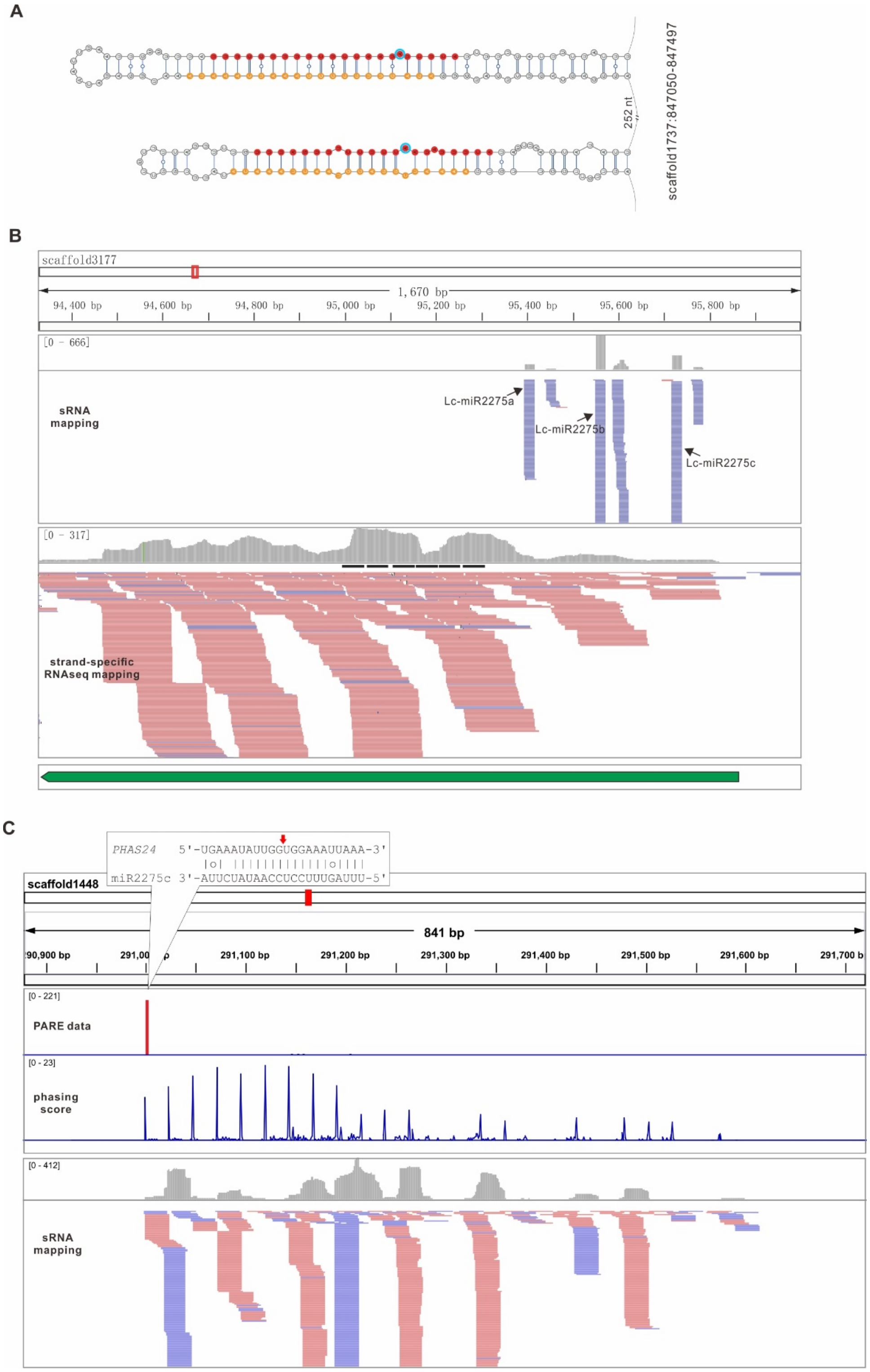

Supplementary Figure 2. miR2275/24-nt phasiRNA pathway components in citrus and cotton, plus polycistronic *MIR2275* precursors in representative monocots and *Amborella*.

A. Mature miR2275 sequences (above) and phasiRNA abundances across different tissues in citrus (below). Tissues in which the phasiRNAs were measured are labeled on the x-axis. In the boxplot, the center line represents the median; box limits are the upper and lower quartiles; whiskers are the 1.5x interquartile range; dots show the scatter of data points. Fruit_WT: fruit from wildtype orange; Fruit_MT: fruit from red-flesh mutant orange.

B. Mature miR2275 sequence (above) and phasiRNA abundance of three 24-*PHAS* loci across different tissues in cotton (below). Every dot represents the phasiRNA abundance of a *PHAS* locus in a given sample. 8H: HT - insensitive (84021), HH: HT - sensitive (H05); HT: High Temperature, NT: Normal temperature; TS: buds of 6–7 mm (tetrad stage), TDS: 9–14 mm (tapetal degradation stage).

C. Polycistronic *MIR2275* loci in representative monocots, with species as indicated above and genomic coordinates indicated below.

D. Polycistronic *MIR2275* loci in *Amborella*.

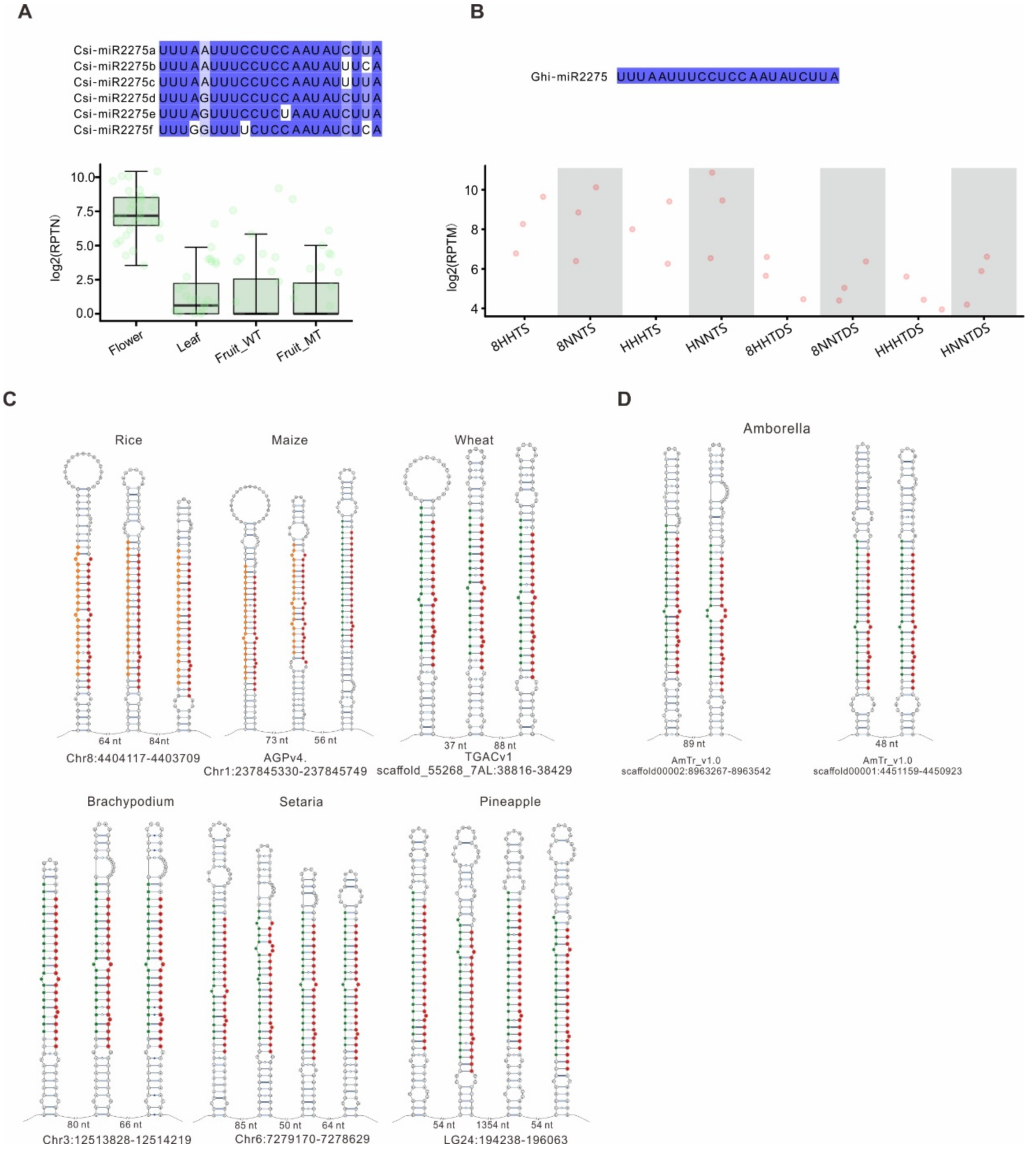

Supplementary Figure 3. A phylogeny of DCL3 and DCL5 homologs, based on the amino acid sequences extracted from the 205 genomes analyzed in this study.
A. A circular cladogram without species details demonstrating that eudicot DCL3, and monocot DCL3 and DCL5, fall into separate, monophyletic groups.
B. An enlarged subtree of eudicot DCL3 proteins, with duplicated DCL3 copies mentioned in the main text marked in red; these represent a more ancient duplication within the eudicot DCL3 branch.

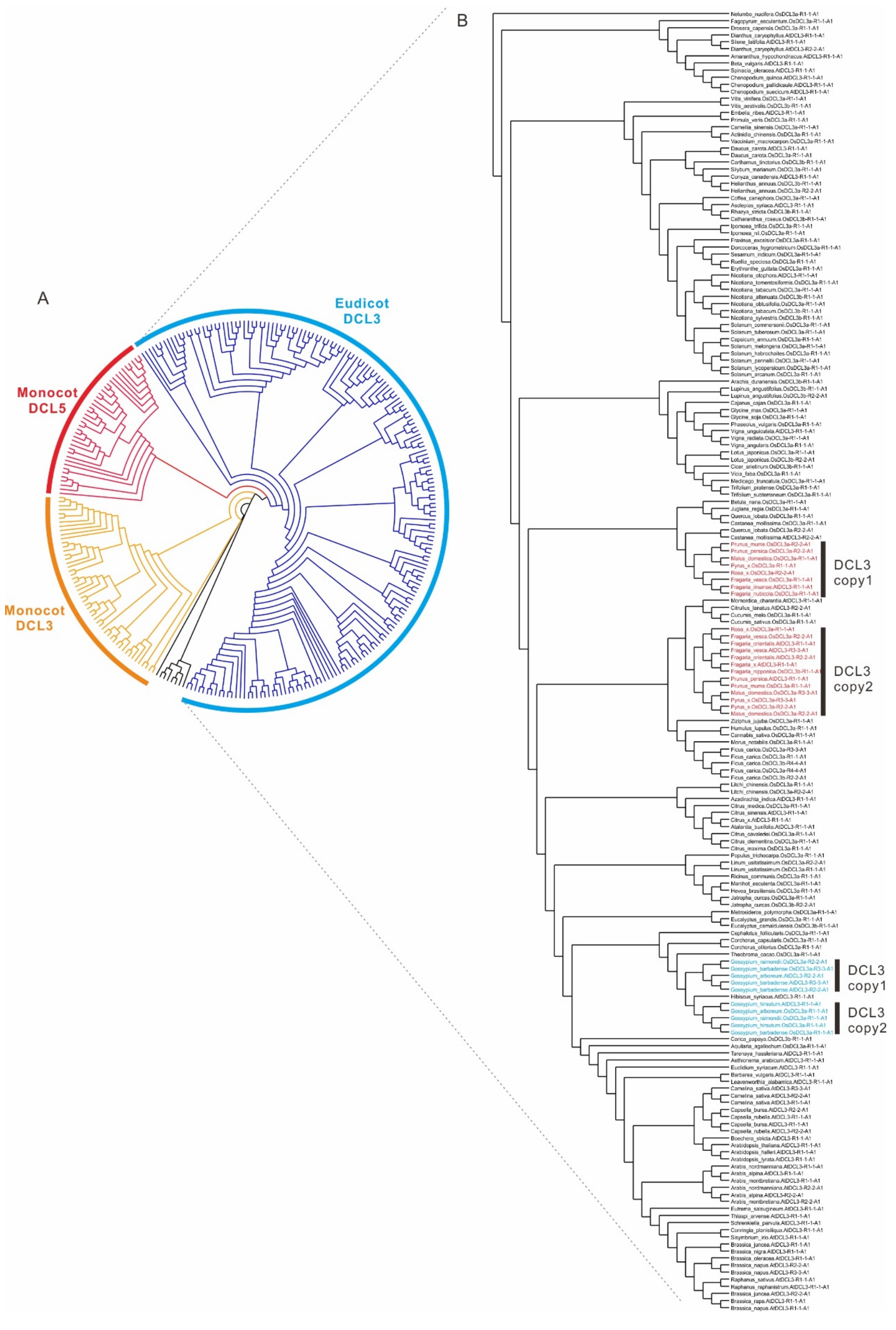

Supplementary Figure 3B, continued (*high resolution, upper portion of the eudicot branch of the tree*)

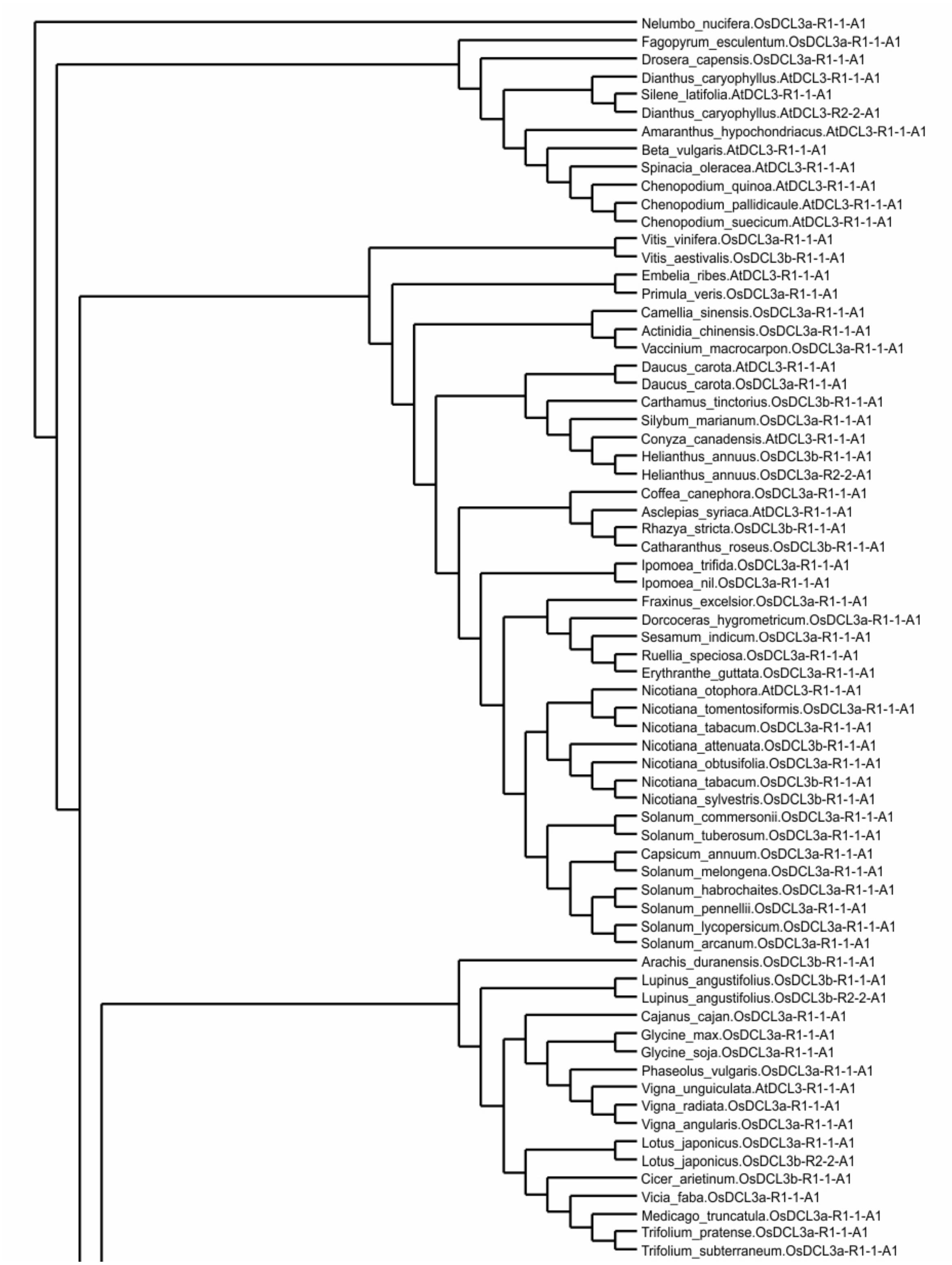

Supplementary Figure 3B, continued (*high resolution, central portion of the eudicot branch of the tree*)

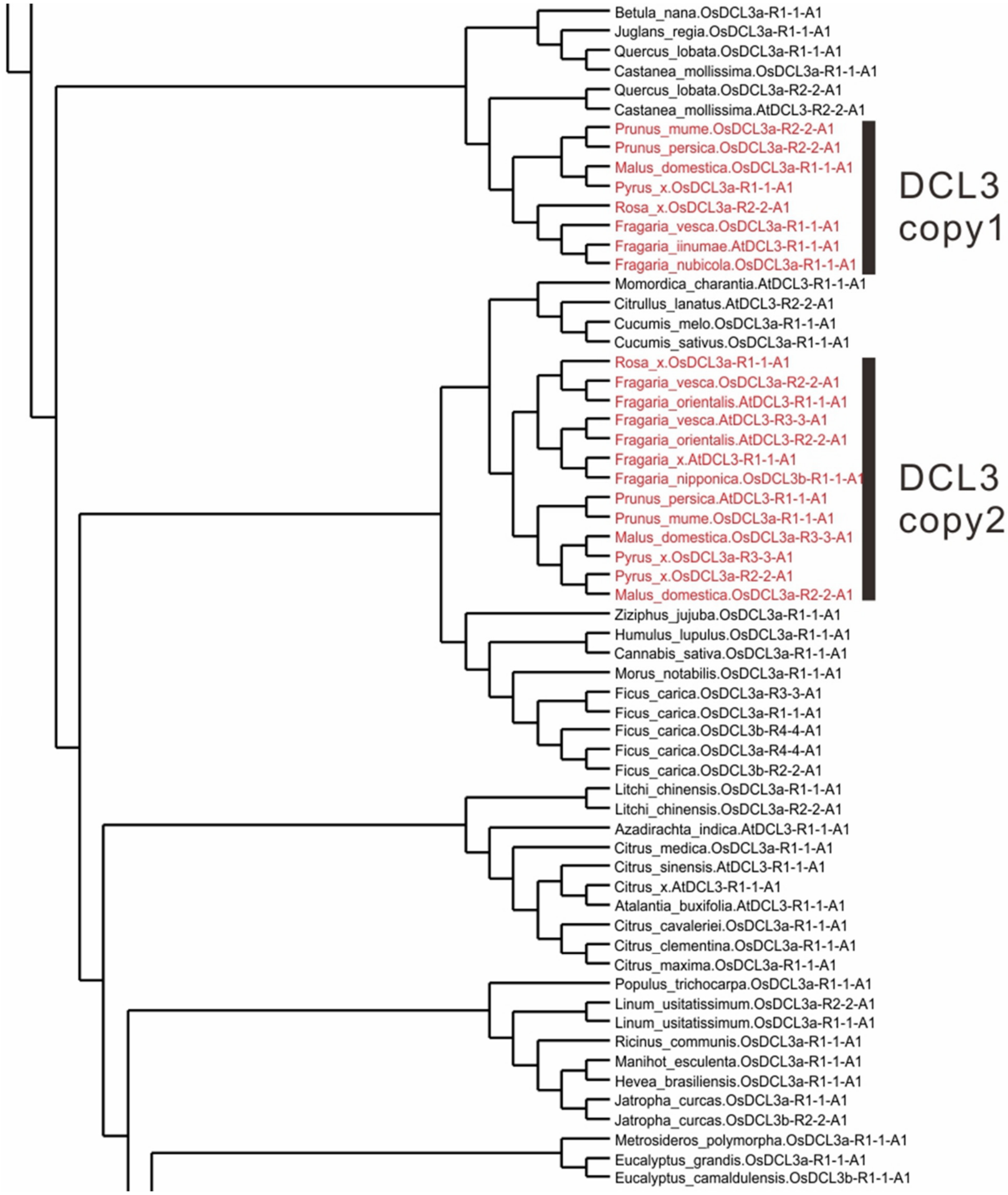

Supplementary Figure 3B, continued (*high resolution, lower portion of the eudicot branch of the tree*)

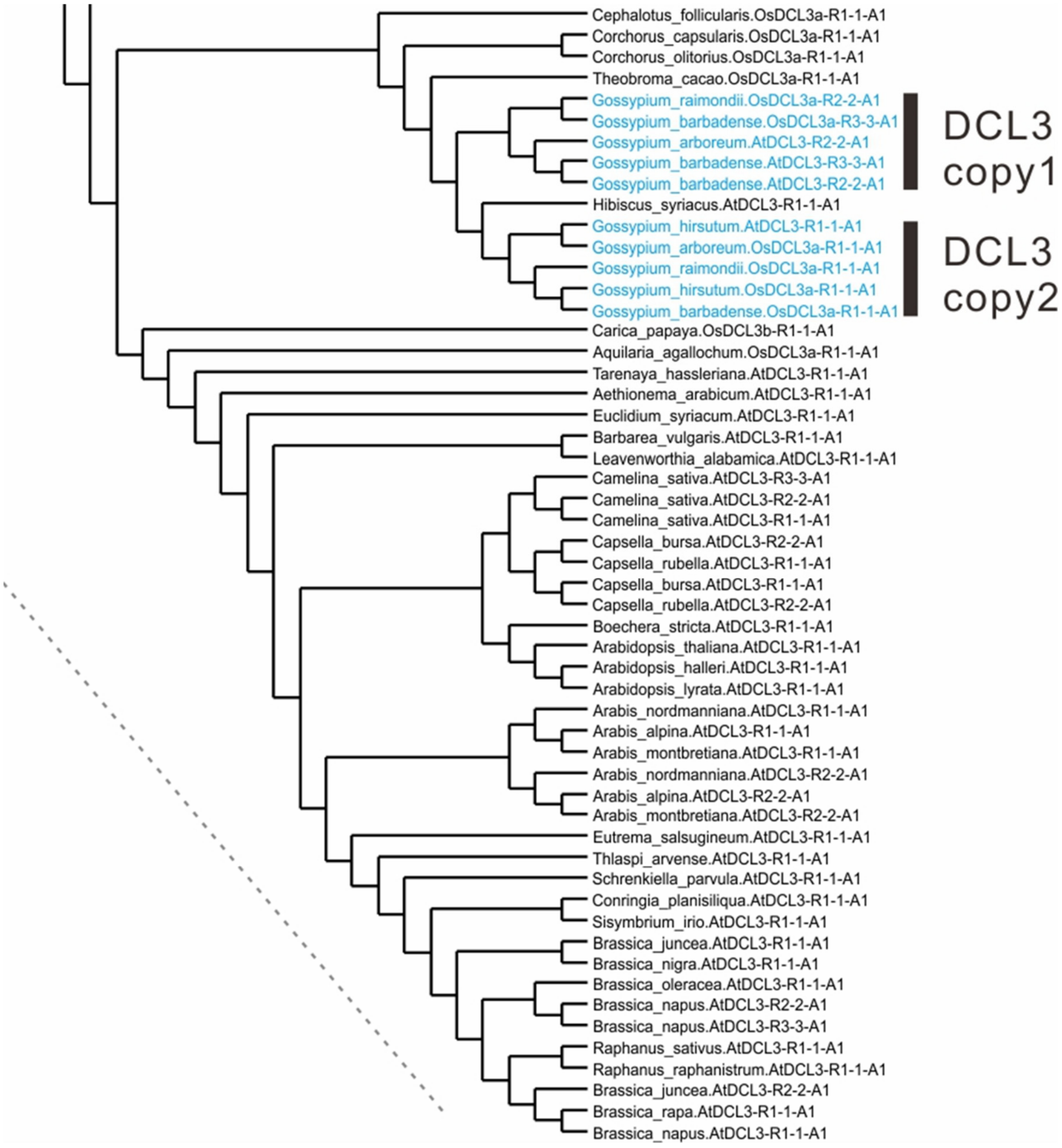

Supplementary Figure 4. Machine learning associates 24-nt reproductive phasiRNAs from diverse species and distinguishes them from 24-nt heterochromatic siRNAs.
Single-nucleotide sequence profiles of position specific base usage comparing 24-nt phasiRNAs (left, panels) and 24-nt hc-siRNAs (right panels) from grasses (rice and maize) and three eudicot plants. The 2000 most abundant phasiRNA or hc-siRNAs were used for this analysis. For grasses, the top most abundant phasiRNAs or hc-siRNAs from the rice and maize data were combined. For each plot, the frequencies of each of the four bases (A, C, G, and U) at each position are indicated as an open circle; markers denoted as small square boxes (highlighted with red dotted circles) represent positions at which a statistically significant (p = 1e-5) base usage distinguishes the nucleotide usage at the same position in the panel at left compared to the panel at right (i.e., comparing 24-nt reproductive phasiRNAs with 24-nt hc-siRNAs within the same species). The methodology was as published (see Patel et al., 2018; DOI: 10.1111/nph.15349)

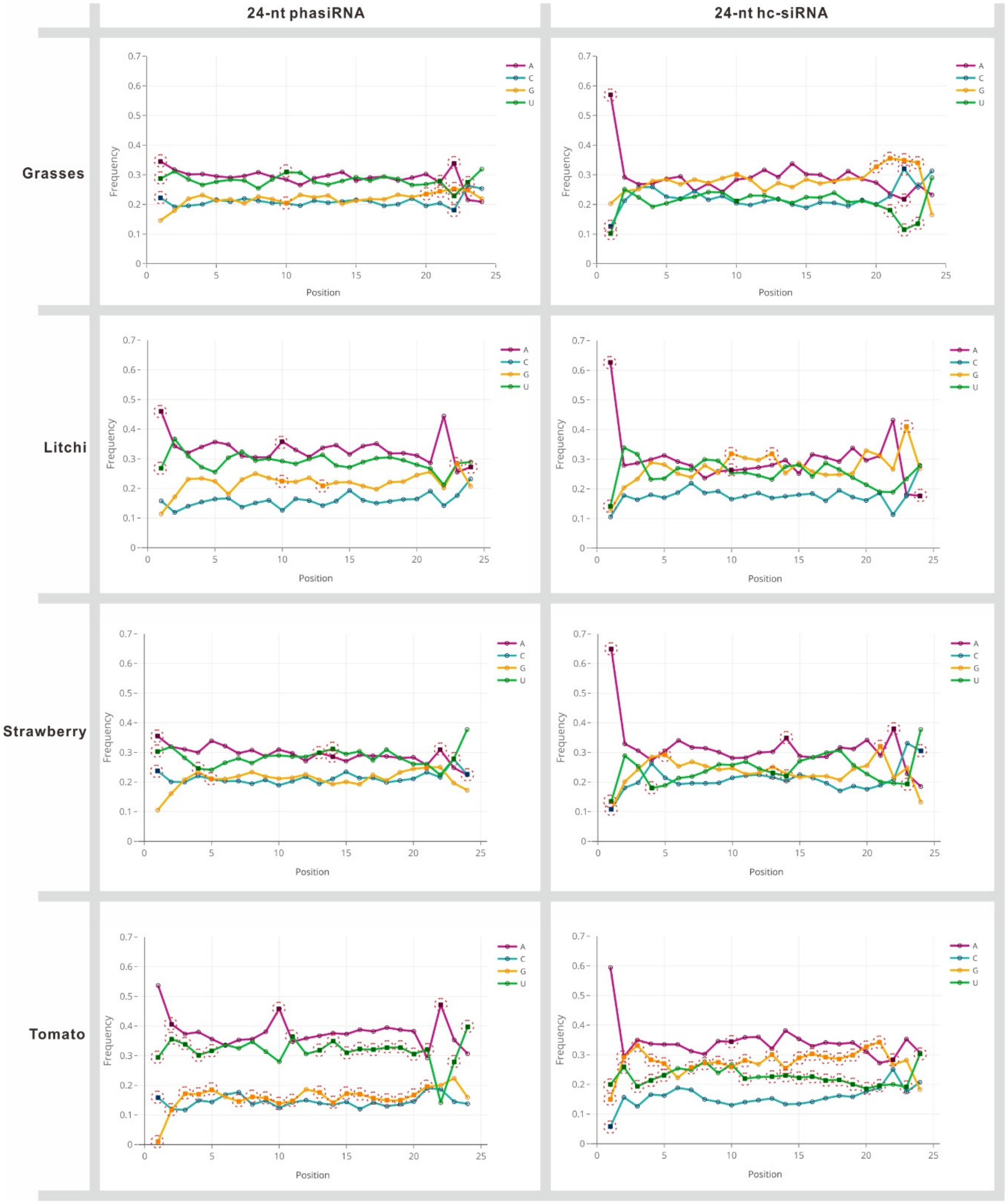

Supplementary Figure 5. 24-nt phasiRNAs found in *Petunia axillaris* and *Petunia inflata*.
A. Accumulation of 24-nt phasiRNAs in *P. axillaris*, with each row denoting a 24-*PHAS* locus.
B. A representative locus generating 24-nt phasiRNAs in *P. axillaris*. Distribution of sRNAs (above), and deduced phasing score (below) are viewed along the coordinates of the 24-*PHAS* locus, with phasiRNA distribution in registers (radar plot) displayed on the left.
C. Accumulation of 24-nt phasiRNAs in *P. inflata*, with each row denoting a 24-*PHAS* locus.
D. A representative locus generating 24-nt phasiRNAs in *P. inflata*. Distribution of sRNAs (above), and deduced phasing score (below) are viewed along the coordinates of the 24-*PHAS* locus, with the phasiRNA distribution in registers (single nucleotide increments) displayed in a radar plot on the left.

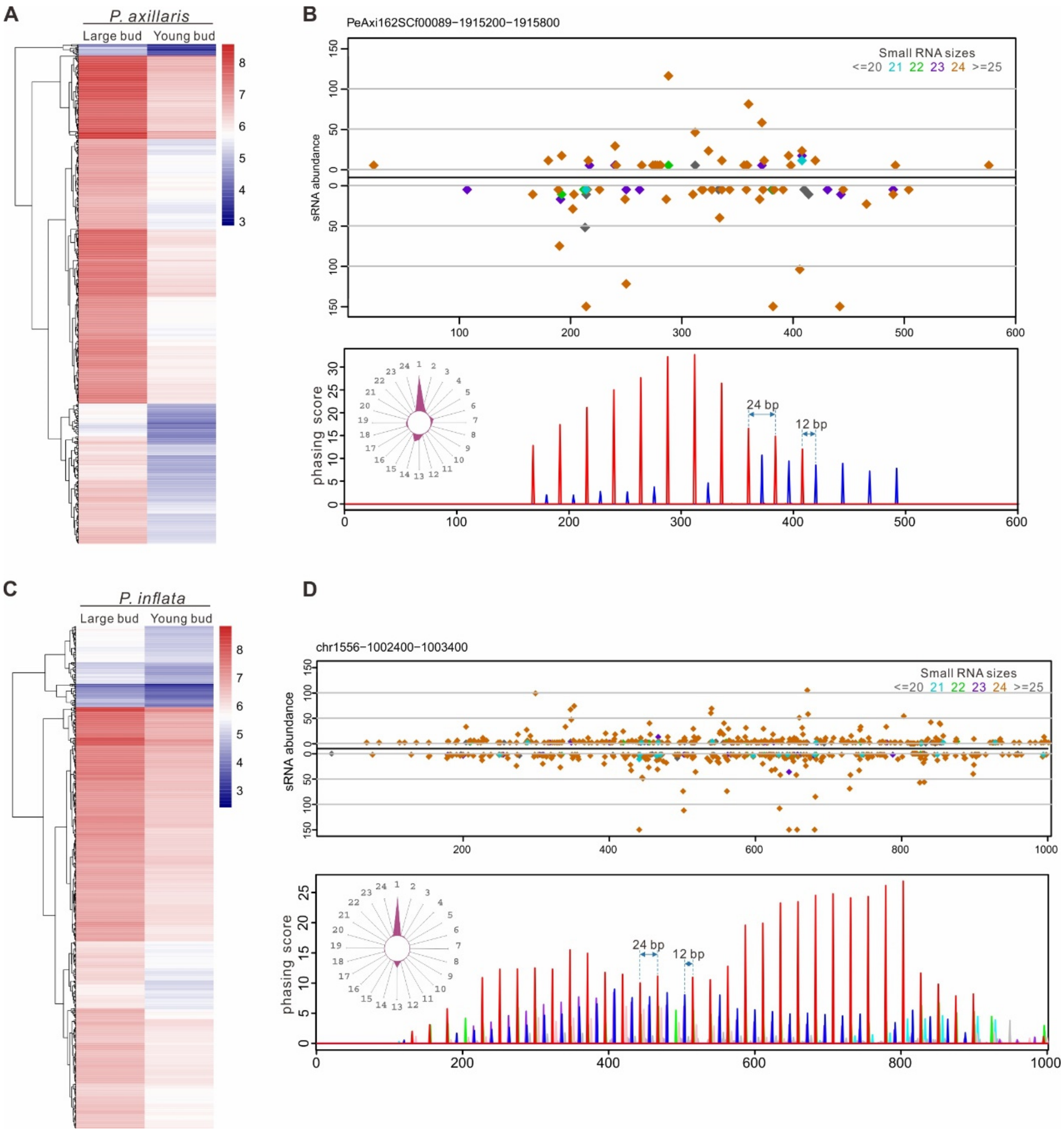

Supplementary Figure 6. Phasing of 24-nt phasiRNAs occurs in both 12- and 24-nt increments. A representative 12-nt phased 24-*PHAS* locus (A) and a representative 24-nt phased 24-*PHAS* locus targeted by miR2275 in rice (B). Distribution of sRNAs (above), and deduced phasing score (below) are viewed along the coordinates of the 24-*PHAS* locus, with phasiRNA distribution in registers displayed in a radar plot on the left (A) or the right (B).

A

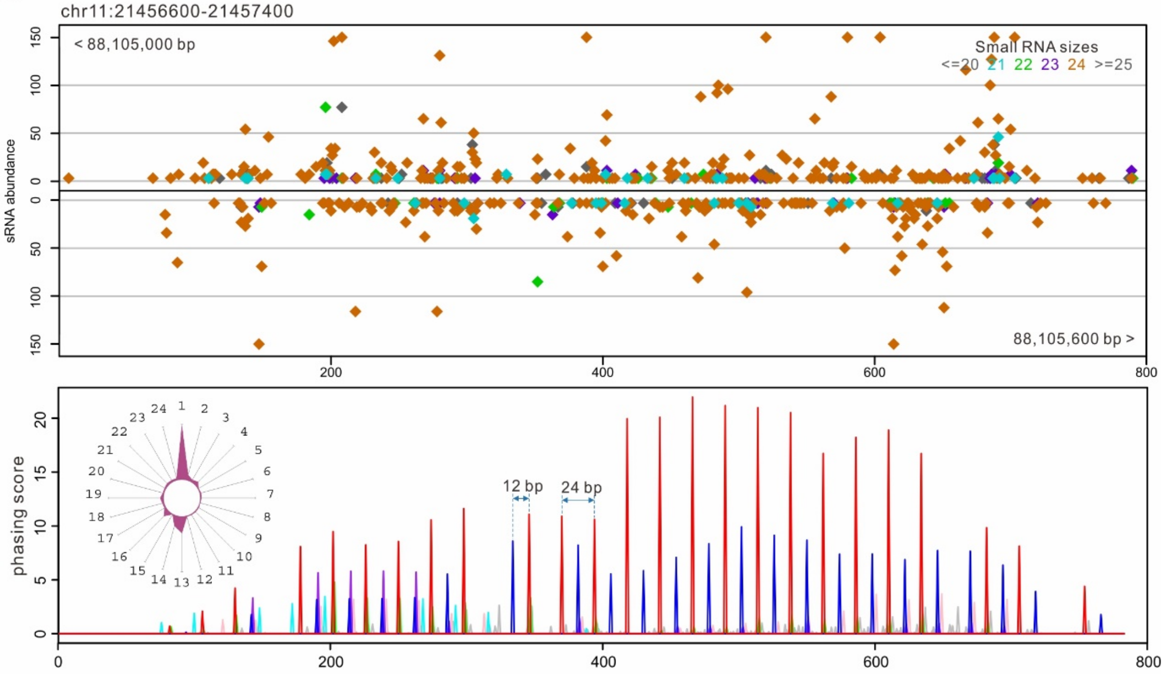

B

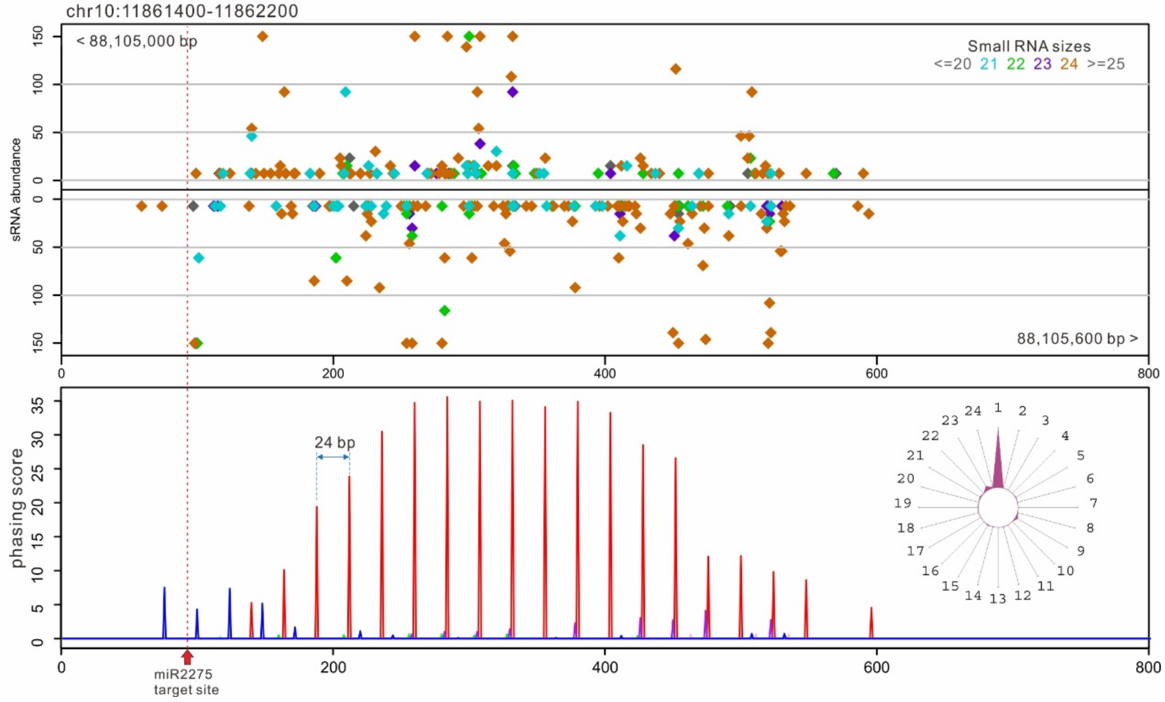

Supplementary Figure 7. A detailed hypothesis for the generation of a 12-nt phasing for 24-nt phasiRNAs. First, the initial 24-nt phase is generated (1). Either strand of a 24-nt phasiRNA duplex can act in *cis* to target the primary transcripts (2) to direct slicing. Subsequently, the cleaved transcripts are converted into double-stranded RNAs via an RNA-dependent RNA polymerase (likely RDR6) (3), which are subsequently processed by Dicer (presumably DCL3 or DCL5, the only plant Dicers known to process 24-nt sRNAs), generating 24-nt phasiRNAs; these phasiRNAs form a secondary 24-nt phase pattern, shifted 12 nt from the primary register of phasing (4). The combination of the primary and secondary phases demonstrates a 12-nt phased pattern when both are mapped to the genome.

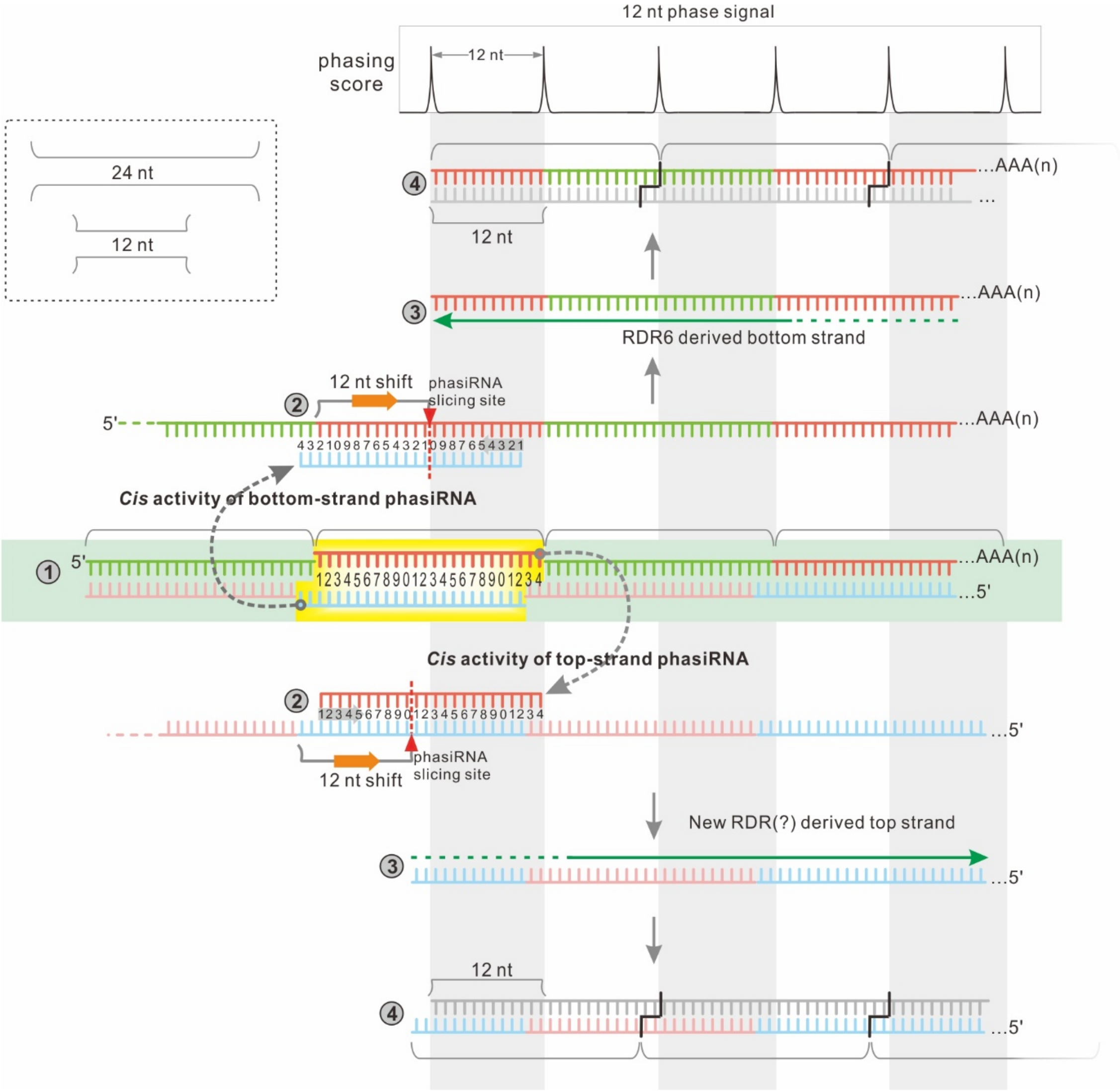

Supplementary Figure 8. Litchi flower buds collected for the evaluation of spatiotemporal expression of 24-nt phasiRNAs.

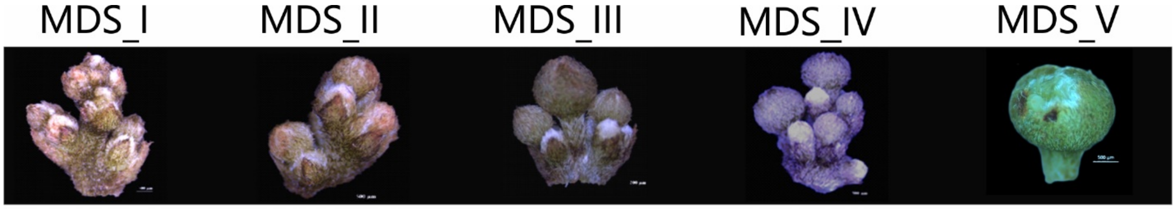

MDS_I: Inflorescences were collected on Feb 29, 2016, approximately 30 DBA (days before anthesis)
MDS_II: Inflorescences were collected on Mar 5, 2016, approximately 25 DBA (days before anthesis)
MDS_III: Inflorescences were collected on Mar 10, 2016, approximately 20 DBA (days before anthesis)
MDS_IV: Inflorescences were collected on Mar 15, 2016, approximately 15 DBA (days before anthesis)
MDS_V: Single flower buds were collected on Mar 20, 2016, approximately 10 DBA (days before anthesis). At this stage, bud heads open slightly.

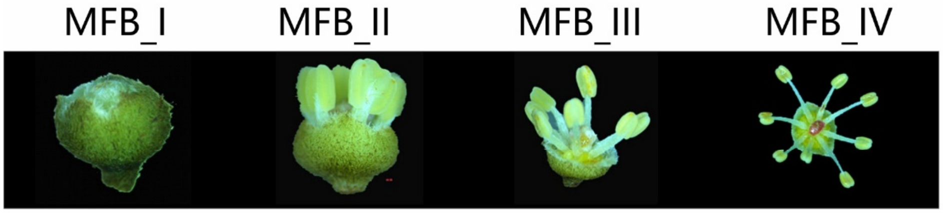

MFB_I: Single flower buds were collected. At this stage, bud heads open wide and anthers can be clearly seen from the opennings.
MFB_II: Single flowers were collected. At this stage, anthers are out and grow faster than the pistil.
MFB_III: Single flowers were collected. At this stage, filaments elongate further and the pistil stops growth.
MFB_IV: Single flowers were collected. At this stage, flowers fully open and the pistil is aborted.

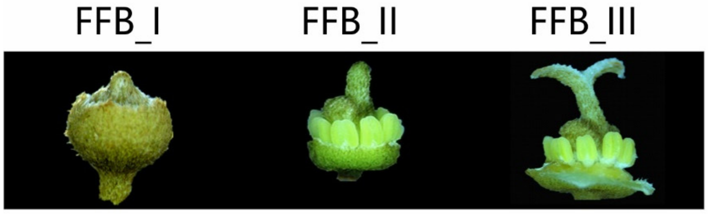

FFB_I: Single flower buds were collected. At this stage, bud heads open wide and the pistil grows out from the top.
FFB_II: Single flowers were collected. At this stage, the pistil grows faster than the anthers, which aggregate at the base.
FFB_III: Single flowers were collected. At this stage, the top of the pistil opens into two parts and anthers stop growth.
