# Supplementary material for: 24-nt reproductive phasiRNAs are broadly present in angiosperms
Source: Nat Commun. 2019 Feb 7;10:627. doi: 10.1038/s41467-019-08543-0 (PMC6367383; doi:10.1038/s41467-019-08543-0)
Supplement: Supplementary file 3 — Description of Additional Supplementary Files [file 41467_2019_8543_MOESM3_ESM.pdf]

Description of Additional Supplementary Files

File Name: Supplementary Data 1
Description: 24-PHAS loci in litchi

File Name: Supplementary Data 2
Description: 24-PHAS loci in orange

File Name: Supplementary Data 3
Description: 24-PHAS loci in grape

File Name: Supplementary Data 4
Description: 24-PHAS loci in strawberry

File Name: Supplementary Data 5
Description: 24-PHAS loci in cotton

File Name: Supplementary Data 6
Description: MIR2275 identified in eudicots

File Name: Supplementary Data 7
Description: The expanded and complete phylogenetic tree showing more detail than the concise version shown in Figure 3D

File Name: Supplementary Data 8
Description: 24-PHAS loci in tomato

File Name: Supplementary Data 9
Description: 24-PHAS loci in Petunia axillaris

File Name: Supplementary Data 10
Description: 24-PHAS loci in Petunia inflata

File Name: Supplementary Data 11
Description: Probes used for in situ hybridizations

File Name: Supplementary Data 12
Description: Public sRNA sequence data used in this study
